# Probiotic Cell-Free Supernatants Exhibited Anti-Inflammatory and Antioxidant Activity on Human Gut Epithelial Cells and Macrophages Stimulated with LPS

**DOI:** 10.1155/2018/1756308

**Published:** 2018-07-04

**Authors:** Stefania De Marco, Marzia Sichetti, Diana Muradyan, Miranda Piccioni, Giovanna Traina, Rita Pagiotti, Donatella Pietrella

**Affiliations:** ^1^Unit of Biochemical Sciences and Health, Department of Pharmaceutical Sciences, University of Perugia, Via del Giochetto, 06122 Perugia, Italy; ^2^Department of Medical Microbiology, Yerevan State Medical University after Mkhitar Heratsi, 2 Koryun Str., 375025 Yerevan, Armenia; ^3^Unit of Food and Nutritional Sciences, Department of Pharmaceutical Sciences, University of Perugia, Perugia, Italy

## Abstract

The incidence of inflammatory bowel disease is increasing all over the world, especially in industrialized countries. The aim of the present work was to verify the anti-inflammatory activity of metabolites. In particular, cell-free supernatants of* Lactobacillus acidophilus, Lactobacillus casei*,* Lactococcus lactis, Lactobacillus reuteri*, and* Saccharomyces boulardii* have been investigated. Metabolites produced by these probiotics were able to downregulate the expression of PGE-2 and IL-8 in human colon epithelial HT-29 cells. Moreover, probiotic supernatants can differently modulate IL-1*β*, IL-6, TNF-*α*, and IL-10 production by human macrophages, suggesting a peculiar anti-inflammatory activity. Furthermore, supernatants showed a significant dose-dependent radical scavenging activity. This study suggests one of the mechanisms by which probiotics exert their anti-inflammatory activity affecting directly the intestinal epithelial cells and the underlying macrophages. This study provides a further evidence to support the possible use of probiotic metabolites in preventing and downregulating intestinal inflammation as adjuvant in anti-inflammatory therapy.

## 1. Introduction

Interest in probiotics and probiotic-based functional foods has grown enormously during the last few years, primarily due to immense health potentials. The internationally endorsed definition of probiotics is live microorganisms that, when administered in adequate amounts, confer a health benefit on the host [1]. It is now well recognized that consumption of probiotic organisms, directly or in the form of their food formulations, can alleviate diseases associated with erratic functioning of human gut besides other chronic life-threatening ailments [2–6]. This explains the efforts made, in recent years, to explore dietary-based interventions to treat chronic diseases (diarrhea and inflammatory bowel diseases), ulcerative colitis, peptic ulcers, Crohn's disease, and constipation, all characterized by compromised gut barrier. Probiotics are included primarily, but not exclusively, in two genera,* Lactobacillus* and* Bifidobacterium* [7]. However, not all candidate probiotics have been proven to be equally efficient.

Several works on the properties and functionality of living microorganisms in food have suggested, indeed, that probiotics play an important role in digestive and respiratory functions, suppression of mutagenesis, tumorigenesis, peroxidation, hypercholesterolemia, or intestinal putrefaction [8–10]. Probiotics could also have a significant effect on alleviation of infectious diseases in children and other high-risk groups [11]. Moreover, several mouse models have demonstrated the effect of probiotics in management of colitis [12, 13]. Oral administration of probiotic foods is known to modulate the host immune response [14]. In particular,* Lactobacillus* is an important member of the probiotic bacteria, which plays an essential role of immunomodulation in the intestinal mucosa [15]. Some studies have shown that they provide a positive effect by promoting the secretion of immunoglobulin IgA and the production of antimicrobial molecules (i.e., bacteriocins), which are capable of inhibiting some intestinal pathogens [16]. Finally, recent studies have shown that metabolites produced by probiotics have antivirulence activity [17].

Probiotics can attach to intestinal epithelial cells (IECs) and modulate their function, directly triggering immune responses by M cells, macrophages, or dendritic cells. A mucous layer covers the intestinal epithelium, segregating microorganisms in the lumen and avoiding their direct contact with cells. Microbial products pass through the mucus and stimulate the epithelial cells [18] but their role in immunomodulation is still largely unknown. Probiotics are usually not in direct contact with macrophages, but when the epithelial barrier is damaged, bacteria and their metabolites can interact with immune cells underlying the epithelium. In this contest, the use of macrophages constitutes an appropriate* ex vivo* human system to study the intracellular cytokine expression pathways [19].

The aim of the present work was to verify whether the metabolites produced by probiotics, which can pass through the mucous, are able to interact with epithelial cells and macrophages inducing an anti-inflammatory state. This study was specifically undertaken with the objective of assessing the health benefits of metabolites produced by five potential probiotic strains (*Lactobacillus acidophilus, Lactobacillus casei*,* Lactococcus lactis, Lactobacillus reuteri*, and* Saccharomyces boulardii)*. Cell-free supernatants (CFS) of probiotic strains have been tested in* in vitro* models with the aim to evaluate their immunomodulatory effects. To confirm the anti-inflammatory effect of probiotics CFS observed for HT29 epithelial cells, we used* ex vivo* human monocytes differentiated in macrophages. The anti-inflammatory activity of CFS in HT-29 human mucus secreting adenocarcinoma cell line and monocyte-derived macrophages (MDM) stimulated with lipopolysaccharide (LPS) has been explored. In this study, we focused on the effect of CFS on the secretion of proinflammatory cytokines such as prostaglandin E2 (PGE-2) and interleukin-8 (IL-8) by HT-29. Moreover, we hypothesized that CFS of chosen probiotics may directly interfere with the host signaling events that drive the intestinal inflammatory response, altering proinflammatory cytokines (IL-1*β*, IL-6, and TNF-*α*) and IL-10 production by MDM. Lastly, we studied the potential of probiotic CFS to exhibit antioxidant properties along with health benefits.

## 2. Materials and Methods

### 2.1. Bacterial Strain and Culture Conditions

*Lactobacillus acidophilus* ATCC 4356,* Lactococcus lactis *ATCC 11454,* Lactobacillus casei *ATCC 334,* Lactobacillus reuteri* ATCC 55148, and* Saccharomyces boulardii *ATCC MYA-796 (Sb48) were purchased from the American Type Culture Collection (ATCC) and LGC Standards S.r.l., Milan, Italy.

One day before the experiment, a colony of* L. acidophilus, L. casei, L. lactis, *and* L. reuteri* has been isolated from each culture and restreaked, separately, onto 14 mL of fresh De Man, Rogosa, and Sharpe (MRS) broth (Sigma-Aldrich, Ottawa, Canada, USA). A single colony of* S. boulardii* was cultivated in Sabouraud broth (Sigma). Microbial suspensions have been incubated for 24 h at 37°C in sterile closed tubes to get microaerophilic conditions. After incubation, probiotic cells were washed in PBS; the number was determined by reading in a spectrophotometer after incubation;* L. acidophilus, L. casei, L. lactis, *and* L. reuteri* reached the concentration of 4.5-5x10^8^/ml; the concentration of* S. boulardii* yeast was about 5x10^7^/ml. Probiotic suspensions were diluted or concentrated to the concentration of 10^8^ CFU/mL

### 2.2. Cell-Free Supernatants (CFS) Production

Cell-free supernatants (CFS) were prepared in Roswell Park Memorial Institute 1640 medium (RPMI 1640, Sigma-Aldrich, Ottawa, Canada, USA). 10^6^ CFU/mL of probiotics cultivated for 24 h in MRS (*Lactobacilli*) or Sabouraud broth (*S. boulardii*) was inoculated in a volume of 14 mL of RPMI 1640 and incubated for around 24 h at 37°C with periodic mixing until suspensions reached the same concentration of 5x10^8^/ml (the concentration was determined by spectrophotometer reading). After incubation, samples were centrifuged at 3000xg for 10 minutes and the pH resulted to be around 6 for lactobacilli and 7 for* S. boulardii*. Supernatants were then sterilized through 0.22 *μ*m cellulose filters (Phenomenex Italia, Castel Maggiore, Italy). CFS were stored at -20°C until use.

### 2.3. HT-29 Treatment

HT-29 human mucus secreting adenocarcinoma cell line (ATCC HTB-38) was cultured in RPMI 1640, supplemented with 10% (v/v) heat-inactivated (56°C/30 min) fetal bovine serum (FBS, Sigma), 100 U penicillin/mL, and 100 *μ*g streptomycin/mL (*c*RPMI), in 25 cm^2^ culture flask at 37°C in an atmosphere of 5% CO_2_.

For determining proinflammatory cytokine production, 4x10^6^ cells/mL HT-29 epithelial cells were seeded to each well of 24-well tissue culture and incubated at 37°C until confluence was reached. The HT-29 monolayers were initially stimulated for 4 h with 100 ng/mL lipopolysaccharide (LPS, Sigma-Aldrich) as previously described [20–22]. After LPS treatment, medium was removed, and cells were incubated in cRPMI with CFS (10% v/v) for additional 18 h at 37°C and 5% CO_2_. The pH of the culture media after CFS addition was measured and resulted to be between 7 and 8. A negative control (untreated sample) was carried out stimulating the cells with noninoculated medium.

Then, samples were centrifuged and supernatants were recovered and stored at -20°C until cytokines analysis.

### 2.4. Monocyte-Derived Macrophages (MDM) Isolation and Stimulation

Heparinized venous blood was obtained from buffy coat of healthy donors who had not taken anti-inflammatory drugs in the previous days, gently provided by Blood Bank of Ospedale della Misericordia of Perugia. All donors have been informed and they signed the consensus form (MO-SIT_06) approved by Ethics Committee CEAS (Comitato Etico Aziende Sanitarie) (Rev. 3 Ottobre 2014) in which they authorize the use of their sample for research studies. Peripheral blood mononuclear cells (PBMCs) were separated by density gradient centrifugation over Ficoll-Hypaque Plus (GE Healthcare Europe GmbH, Milan, Italy), recovered, washed twice, and suspended in cRPMI.

Monocyte-derived macrophages (MDM) were obtained from PBMC. Following isolation, PBMCs were seeded into 75 cm^2^ flask (Corning Incorporated, Corning, NY) in serum-free RPMI 1640 and incubated for 1-2 h at 37°C and 5% CO_2_ in order to allow monocyte adhesion. After incubation, adherent peripheral blood monocytes were recovered with a cell scraper (Falcon, Oxford, California) and washed twice. 2×10^5^ MDM/mL was seeded in cRPMI in 24-well plates at 37°C in a humidified atmosphere containing 5% CO_2_. Cells were treated with CFS (10% v/v) before or after LPS (1 *μ*g/mL) stimulation. 25 *μ*g/mL dexamethasone (Sigma-Aldrich) was used as a positive control. A negative control (untreated sample) was carried out stimulating the cells with noninoculated medium. After treatment, samples were centrifuged and supernatants were recovered and stored at -20°C until cytokines analysis.

### 2.5. Cell Viability

Viability of HT-29 cells and MDM was tested by the determination of the cell ATP level by ViaLight® Plus Kit (Lonza, Italy). The method is based upon the bioluminescent measure of ATP which is present in all metabolically active cells. The bioluminescent method utilizes the luciferase, an enzyme that catalyses the formation of light from ATP and luciferin. The emitted light intensity is linearly related to the ATP concentration and it is measured using a luminometer.

After treatments with CFS, Cell Lysis Reagent was added to each well to extract ATP from cells. Next, after 10 minutes, the AMR Plus (ATP Monitoring Reagent Plus) was added and after 2 more minutes the luminescence was read using a microplate luminometer (TECAN). Results were expressed as percentage of Relative Luminescence Unit (RLU). RLU of untreated cells at time 0 has been subtracted.

### 2.6. Cytokines Determination

To analyze PGE-2, IL-8, IL-1*β*, IL-6, TNF-*α*, and IL-10, supernatants were collected and stored at -20°C until analysis. The concentration of secreted cytokines and chemokine was determined in the supernatants of cells by ELISA (U-CyTech biosciences, Utrecht, Netherlands) according to the manufacturer's guidelines.

### 2.7. Free Cell Antioxidant Assay

The antioxidant activity of cell-free probiotics supernatants was evaluated by using the 2,2-diphenyl-1-picrylhydrazyl (DPPH, Sigma-Aldrich) free radical scavenging assay as described previously [20] with some modifications. This widely used discoloration method was first described by Blois [21] and is based on the premise that a hydrogen donor is an antioxidant.

Antioxidants are able to reduce the free, stable, and purple-coloured DPPH radical to the yellow-coloured diphenylpicrylhydrazine, which is monitored by using a colorimeter [22].

CFS were diluted in ethanol at different concentrations (1, 5, and 10% v/v) and added to an ethanol solution of DPPH (25 *μ*g/mL). After 30 min of reaction at room temperature in the dark, the absorbance of each solution was read at 517 nm in a spectrophotometer (TECAN). The mixture of ethanol and sample was used as blank. The control solution was prepared by mixing ethanol and DPPH radical solution. Ascorbic acid (Sigma-Aldrich), at concentration of 100 *μ*g/mL, was used as a positive control.

The percentage of inhibition was calculated using the following formula:

(1) DPPH  scavenging  activity %=100−A  sample−A  blank×100A  control

where A sample is absorbance of the sample after 30 min of reaction, A blank is absorbance of the blank, and A control is absorbance of the control.

Each measure was performed in duplicate in three individual experiments.

### 2.8. Antioxidant Activity on Ex Vivo Human Neutrophils

Neutrophils were obtained from PBMC. PBMCs were isolated from fresh buffy coats as described previously. Neutrophils were isolated by density gradient centrifugation as previously described [23]. Erythrocytes were removed by ammonium-chloride-potassium lysing buffer (ACK buffer). Following isolation, the cells were resuspended in cRPMI. Antioxidant activity was evaluated by chemiluminescence assay [23] with minor modifications. Chemiluminescence measurements were performed in a final volume of 0.25 mL. 50 *μ*L of luminol (0.28 mM) and 50 *μ*L of different concentrations of the CFS were added to 100 *μ*L of neutrophil solution (1.25×10^6^ cells/mL) and the mixture was incubated for 3 minutes at 37°C. The cells were then stimulated with 50 *μ*L of 10^−7^ M phorbol-12-myristate-13-acetate (PMA, Sigma-Aldrich). The chemiluminescence produced by the cells was monitored for 20 minutes in a luminometer (Tecan), in which the light output was recorded as RLU (Relative Luminescence Unit). Each measure was performed in triplicate.

### 2.9. Statistical Analysis

Results are given as mean ± standard deviation (SD). Significance was tested by means of a Student's two-tailed* t*-test.* P*<0.05 was considered significant.

## 3. Results

### 3.1. Anti-Inflammatory Activity on Epithelial Cells

**Figure 1:**
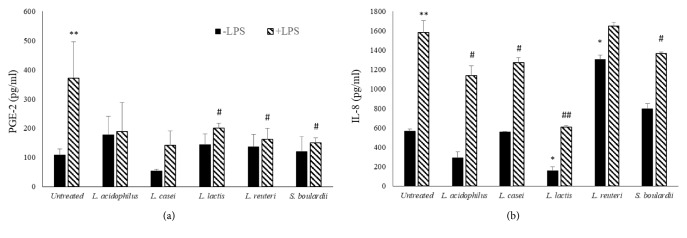
**PGE-2 and IL-8 production in LPS-stimulated HT29 cells**. PGE-2 (a) and IL-8 (b) production in HT-29 cell line challenged with LPS (100 ng/mL) for 4 h and treated with CFS (10% v/v) for 18 h. Results have been obtained from three independent experiments. *∗P*<0.05 and *∗∗P*<0.01 (CFS-treated HT-29* versus* untreated cells); #*P*<0.05 and ##*P*<0.01 (LPS/CFS-treated HT-29* versus* LPS treated HT-29).

The modulation of PGE-2 and IL-8 production in human intestinal epithelial cell lines (HT-29) stimulated by LPS and treated with supernatants of selected probiotics growth in RPMI (10 % v/v) was analyzed (Figure 1).

Under the stimulus with proinflammatory molecules such as LPS, HT-29 cells produce a greater amount of prostaglandin E2 (PGE-2) and IL-8 cytokine. In this case,* L. lactis, L. reuteri*, and* S. boulardii* supernatants were able to significantly reduce the production of PGE-2 (Figure 1(a)).

Among the five probiotics tested, only the supernatant of* L. lactis* is able to reduce the basal production of IL-8 (Figure 1(b)), while LPS-induced IL-8 production was reduced by the supernatants of* L. acidophilus, L. casei, L. lactis, *and* S. boulardii*. Overall,* L. lactis* showed the best anti-inflammatory activity on HT-29 cells. The decrease of IL-8 production is not related to viability of cells, which was not affected by LPS stimulation and/or CFS supernatants treatment (data not shown).

### 3.2. Anti-Inflammatory Activity on Human Macrophages

To confirm the anti-inflammatory effect of probiotics CFS observed for HT-29 epithelial cells, we used* ex vivo* human monocytes differentiated in macrophages.

Human MDM were first pretreated with CFS and then stimulated with LPS for verifying their protective anti-inflammatory activity. In parallel experiments, macrophages were first stimulated with LPS and then treated with CFS to study the ability of probiotics to downregulate the inflammatory response.

**Figure 2:**
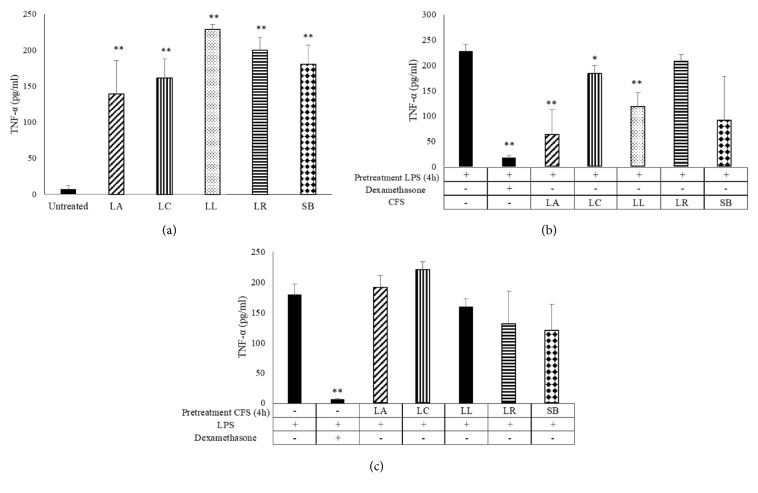
**TNF-** **α** ** production in LPS-stimulated monocyte-derived macrophages (MDM)**. TNF-*α* production in MDM challenged with probiotic CFS (a) and LPS (1 *μ*g/mL) for 4 h and then with CFS (10%) (b) or pretreated with CFS and then stimulated with LPS (c) as indicated in the figure has been tested by ELISA. Results are expressed as mean ± SD of six determinations obtained by six independent subjects. LA:* L. acidophilus*; LC:* L. casei*; LL:* L. lactis*; LR:* L. reuteri*; SB:* S. boulardii*. *∗P*<0.05 and *∗∗P*<0.01, (CFS-treated MDM* versus* untreated cells or CFS-treated MDM* versus* LPS treated cells).

All probiotic CFS have induced by themselves TNF-*α* production (Figure 2(a)); instead, when MDM were challenged with inflammatory stimulus, such as LPS, a downregulation of TNF-*α* production has been observed in presence of CFS of* L. acidophilus, L. casei,* and* L. lactis *(Figure 2(b)). No modulation of the cytokine production has been detected when cells were pretreated with CFS and then stimulated with LPS (Figure 2(c)).

**Figure 3:**
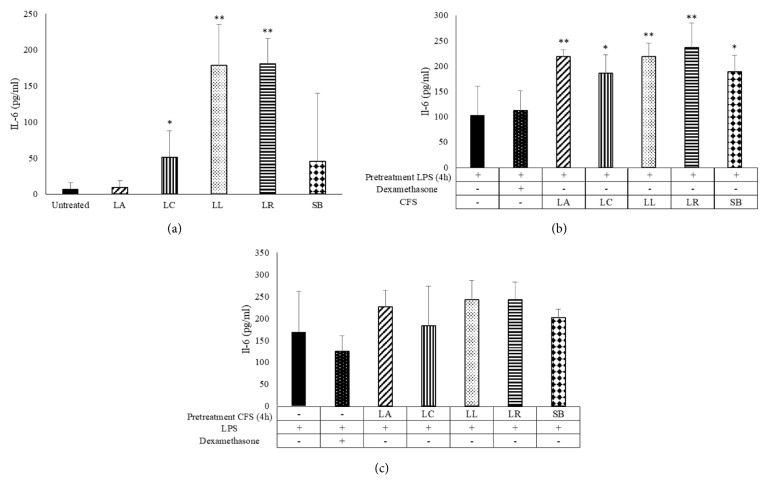
**IL-6 production in LPS-stimulated monocyte-derived macrophages (MDM)**. IL-6 production in MDM challenged with probiotic CFS (a) and LPS (1 *μ*g/mL) for 4 h and then with CFS (10%) (b) or pretreated with CFS and then stimulated with LPS (c) as indicated in the figure has been tested by ELISA. Results are expressed as mean ± SD of six determinations obtained by six independent subjects. LA:* L. acidophilus*; LC:* L. casei*; LL:* L. lactis*; LR:* L. reuteri*; SB:* S. boulardii. ∗P*<0.05 and *∗∗P*<0.01 (CFS-treated MDM* versus* untreated cells or CFS-treated MDM* versus* LPS treated cells).

IL-6 secretion is induced by* L. casei, L. lactis*, and* L. reuteri* CFS but it is not stimulated by* L. acidophilu*s and* S. boulardii *(Figure 3(a)). Contrary to what has been observed for TNF-*α*, all probiotic CFS increased IL-6 production in LPS prestimulated MDM (Figure 3(b)). Again, pretreatment with CFS metabolites does not alter the MDM response to the inflammatory stimulus (Figure 3(c)).

**Figure 4:**
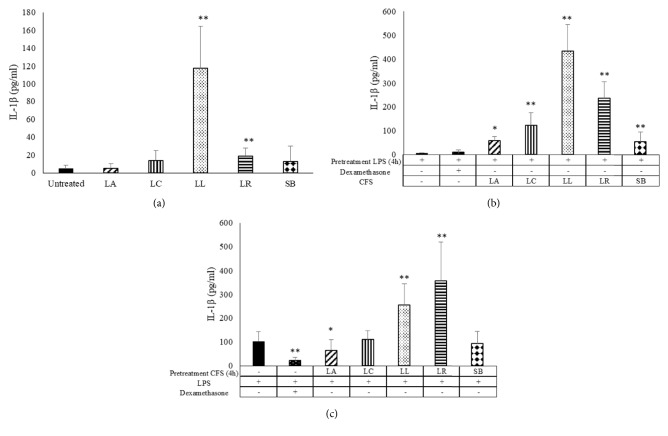
**IL-1** **β** ** secretion by LPS-stimulated monocyte-derived macrophages (MDM)**. IL-1*β* secretion by MDM challenged with probiotic CFS (a) and LPS (1 *μ*g/mL) for 4 h and then with CFS (10%) (b) or pretreated with CFS and then stimulated with LPS (c) as indicated in the figure has been tested by ELISA. Results are expressed as mean ± SD of six determinations obtained by six independent subjects. LA:* L. acidophilus*; LC:* L. casei*; LL:* L. lactis*; LR:* L. reuteri*; SB:* S. boulardii*. *∗P*<0.05 and *∗∗P*<0.01, (CFS-treated MDM* versus* untreated cells or CFS-treated MDM* versus* LPS treated cells).

In addition to TNF-*α* and IL-6 determination, the effect of metabolites of probiotic CFS on IL-1*β* secretion has been investigated. In our experimental model, only* L. lactis* and* L. reuteri* CFS were able to stimulate the IL-1*β* secretion by untreated MDM (Figure 4(a)) or MDM pretreated with CFS (Figure 4(c)). Interestingly,* L. acidophilus *CFS is able to downregulate the secretion of IL-1*β* by MDM induced by LPS (Figure 4(c)). All probiotic CFS tested were able to induce the secretion of the cytokine (Figure 4(b)).

**Figure 5:**
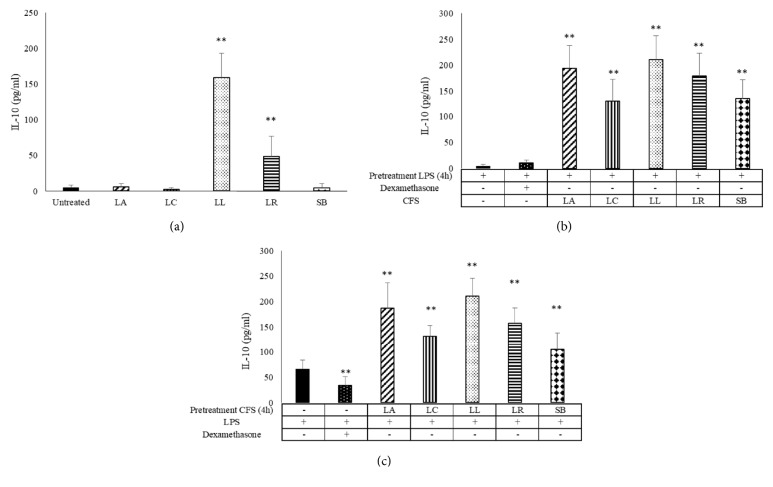
**IL-10 production in LPS-stimulated monocyte-derived macrophages (MDM)**. IL-10 production in MDM challenged with probiotic CFS (a) and LPS (1 *μ*g/mL) for 4 h and then with CFS (10%) (b) or pretreated with CFS and then stimulated with LPS (c) as indicated in the figure has been tested by ELISA.. LA:* L. acidophilus*; LC:* L. casei*; LL:* L. lactis*; LR:* L. reuteri*; SB:* S. boulardii*. Results are expressed as mean ± SD of six determinations obtained by six independent subjects. *∗∗P*<0.01 (CFS-treated MDM* versus* untreated cells or CFS-treated MDM* versus* LPS treated cells).

In addition to the innate proinflammatory cytokines, such as TNF-*α*, IL-1*β*, IL-6, and IL-8, particular attention has been focused on anti-inflammatory IL-10 production. As observed for IL-1*β* basal production, only* L. lactis* and* L. reuteri* are able to stimulate the IL-10 secretion (Figure 5(a)). Data obtained from our study showed that supernatants of all probiotics induced significantly IL-10 secretion by MDM before or after LPS stimulus challenge (Figures 5(b) and 5(c)). When macrophages are in inflammatory state induced by pretreatment with LPS, all CFS tested upregulated the secretion of this anti-inflammatory cytokine (*P*<0.05).

### 3.3. Antioxidative Activity

**Figure 6:**
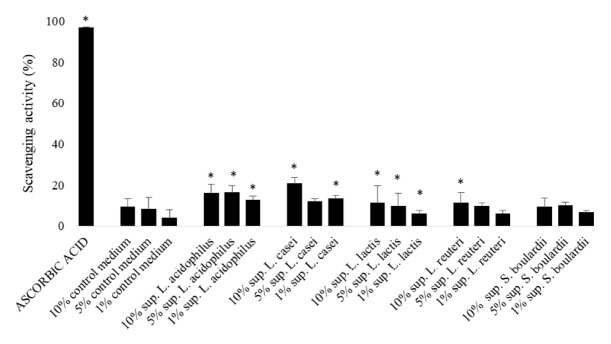
**Antioxidant activity of CFS of probiotics. **Results are expressed as % of DPPH scavenging activity. Data represent the mean ± SD of three independent experiments performed in triplicate. Statistical analysis of raw data was performed by* t-*test, *∗P*<0.05 (CFS* versus* control medium).

Supernatants of* L. acidophilus, L. casei, L. lactis, *and* L. reuteri *showed a slight significant dose-dependent radical scavenging activity with respect to the control consisting in pure medium without CFS (Figure 6). The highest antioxidant activity has been observed for* L. casei *(20.8%).

**Figure 7:**
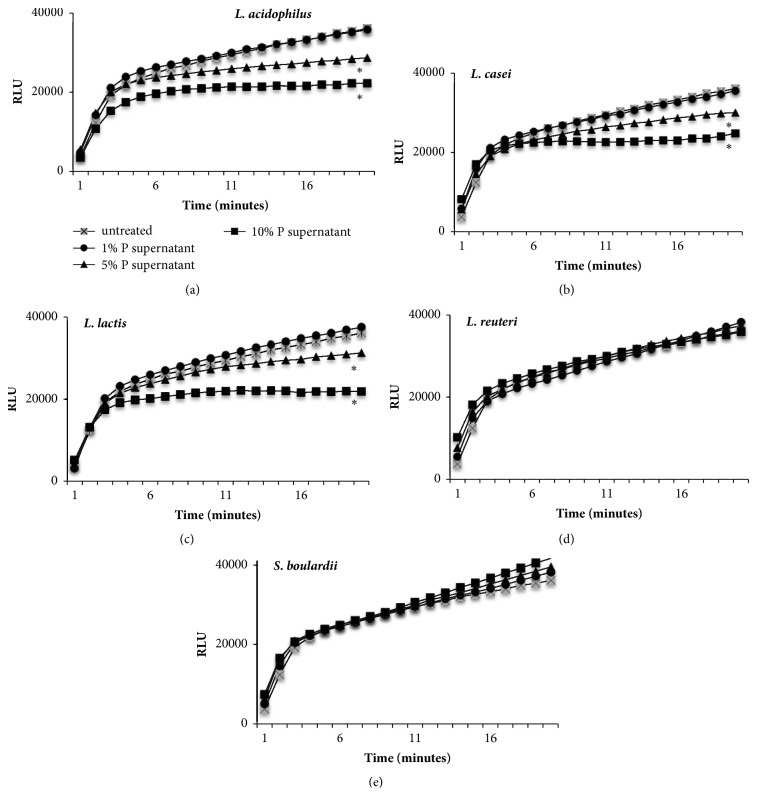
**Probiotic CFS effect on* in vitro* neutralization of ROS produced by PMA-stimulated human neutrophils**. Supernatants of* L. acidophilus* (a),* L. casei* (b),* L. lactis *(c),* L. reuteri* (d), and* S. boulardii* (e) have been tested on ROS production by human neutrophils challenged with PMA for 20 minutes. Results are expressed as Relative Luminescence Unit (RLU). Data represent the mean of two independent experiments performed in triplicate.* t-*test, *∗P*<0.05 (CFS-treated neutrophils* versus* untreated neutrophils).

Furthermore, the antioxidant activity of supernatants was tested in the polymorphonuclear neutrophils from healthy human donors. Supernatants of* L. acidophilus *(Figure 7(a))*, L. casei *(Figure 7(b)), and* L. lactis *(Figure 7(c)) decreased the neutrophil hydrogen peroxide production in concentration-dependent manner, confirming data obtained from DPPH radical scavenging test. The highest degree of inhibition was detected at concentration of 10% (v/v). No effect has been observed for* L. reuteri* and* S boulardii* CFS (Figures 7(d) and 7(e)).

## 4. Discussion

Inflammation is the mark of many inflammatory disorders. The intestinal immune system has developed a number of distinct mechanisms to dampen mucosal immunity and to optimize the responses against microbiota.

The intestinal epithelium is both a barrier and a site of absorption of the luminal contents of the bowel. During intestinal inflammation, the functions of the intestinal epithelium and its permeability are affected: intestinal epithelial cells participate in the initiation and regulation of the mucosal immune response to bacteria by interacting with immune cells of the gut associated lymphoid tissue,* lamina propria* lymphocytes, and intraepithelial lymphocytes [24]. In fact, IECs not only are target of inflammatory mediators but also actively participate in the regulation of inflammatory reactions [25]. The intestinal epithelial monolayer consists of several subsets of epithelial cells which cooperatively constitute a physical and biochemical network for the maintenance of the homeostasis between the body and the luminal environment [26, 27].

Probiotic strains, studied in this work, were chosen exactly based on their immune-modulatory activity.* In vitro* efficacy of* L. acidophilus* LA-14 to modulate the human anti-inflammatory immune response has previously been investigated [28]; there is a preliminary evidence that probiotic supplementation of* L. casei* Shirota improved immunological parameters and reduced key inflammatory cytokine markers [29].* L. lactis *NCDO 2118 has been used in the treatment of inflammatory bowel disease (IBD), since it was able to reduce IL-1*β*-induced IL-8 secretion in Caco-2 cells [30].* In vitro* study demonstrated that* L. reuteri* CRL1098 soluble factors significantly reduced the production of proinflammatory mediators (NO, COX-2, and Hsp70) and proinflammatory cytokines (TNF-*α* and IL-6) caused by the stimulation of macrophages with LPS [31].* S. boulardii* exerted an anti-inflammatory effect by producing a low molecular weight soluble factor in intestinal epithelial cells and monocytes [32]. Low molecular weight factors have been studied for the effects on cytokine expression in other reports as well [33].

PGE-2 is one of the major mediators of inflammation in colorectal cancer (CRC) development and progress [34], same as IL-6, which has been considered as a key regulator of CRC development [35] and increased quantities of plasma IL-6 were correlated with a poor prognosis in a variety of cancers, including colon cancer [36]. Our results are in line with those obtained from Otte et al. (2009) who have demonstrated how probiotics are able to downregulate the production of PGE-2 and cyclooxygenase-2 [37]. In contrast, a study on human gingival fibroblasts showed that the supernatants of two mixed* L. reuteri* strains stimulated the production of PGE-2, suggesting that bacterial products secreted from* L. reuteri* might play a role in the resolution of oral inflammation [38]. These contrasting results suggest that probiotics can have distinct effects on different epithelial cells reflecting the peculiar environmental sites.

The chemokine IL-8 plays a very important role in the recruitment of other immune cells during an inflammatory response [39]. Different cell types, such as mononuclear phagocytes, endothelial cells, fibroblasts, and epithelial cells, can produce IL-8. Rocha-Ramírez* et al.* [40] demonstrated that the chemokine IL-8 is produced during the early stages of the interaction of* Lactobacillus* cells and macrophages (i.e., within 6 h of stimulation), and this response was sustained for 24 h at much higher levels (>2000 pg/mL) than other cytokines productions analyzed in this study. The mechanism of induction of IL-8 in IECs is not known. However, an increasing amount of evidence suggests that IL-8 has an important role in the pathogenesis of IBD [41, 42]. Our results appear to be consistent with the findings of several other investigators who similarly reported considerable reduction in IL-8 expression with probiotic treatment under* in vitro* studies, using different strains of probiotics and inflammatory agents [32, 43].

Circulating monocytes can be recruited to the tissue, where they start to differentiate into macrophages (MDM) under the action of local factors. Once differentiated, MDM become long-lived cells and develop specialized functions in tissue inflammation and maintaining tissue homeostasis. They are antigen-presenting cells that distribute to peripheral tissues where they play multiple roles in diverse physiological processes including host defence, inflammation resolution, and tissue remodelling [44]. A very recent research has shown that heat-inactivated cells of* Lactobacillus* (*Lactobacillus rhamnosus* GG,* L. rhamnosus* KLSD,* L. helveticus* IMAU70129, and* L. casei* IMAU60214) induced MDM to produce early proinflammatory cytokines such as IL-8, TNF-*α*, IL-12p70, and IL-6 between 6 and 24  h after the treatment began [40]. Rocha-Ramírez* et al.* concluded that each one of the strains of* Lactobacillus* tested induced a strong inflammatory response in macrophages. Our study shows that metabolites of probiotics, unlike live or inactivated cells, can have a different immunomodulatory effect.

When MDM were treated with LPS, before CFS stimulation, all probiotics induced the IL-1*β* secretion, suggesting that probiotics metabolites behave as a second signal required for inflammasome activation. In fact, the secretion of mature IL-1*β* is different from that of IL-6 and TNF-*α*, because NF-*κ*B activation ends with the production of the pro-IL-1*β* proteins that cannot be released immediately from the cells. The maturation of IL-1*β* requires the activation of multiprotein complex consisting of pro-caspase-1 enzyme and adaptor molecules (NLRP3), named inflammasome [45, 46]. Interestingly, CFS of* L. acidophilus* pretreatment is able to downregulate the secretion of IL-1*β* by MDM induced by LPS. This result further highlights the different effects of metabolites produced by probiotics by emphasizing their different activity.

IL-10 is an anti-inflammatory cytokine that downregulates proinflammatory cascade. IL-10 has been shown to play a role in chronic gastrointestinal problems, and its modulation by probiotic bacteria has been observed in patients with ulcerative colitis and IBD [47].

IL-10 is of particular therapeutic interest in IBD, since it has been shown that IL-10^−/−^ mice spontaneously develop intestinal inflammation characterized by discontinuous transmural lesions [48]. More recently, it has been shown that probiotic strains offer the best protection against* in vivo *colitis in animal models, hence displaying an* in vitro *potential to induce high levels of IL-10 and low levels of the inflammatory cytokine IL-12 [49].

In our knowledge, there are few studies that analyze the anti-inflammatory activity of CFS. Most studies reported immunomodulation of probiotics on macrophages but in all of them the experimental model consists in the direct contact of macrophages with the bacterial cells. However, a recent research has investigated the anti-inflammatory effects of CFS from* L. acidophilus* and* L. rhamnosus* GG in PMA-differentiated THP-1 cells. Results indicate that CFS from* L. acidophilus* and* L. rhamnosus* GG possess anti-inflammatory properties and can modulate the inflammatory response as observed in our experimental model [50]. Another study of Bermudez-Brito* et al*. has shown that probiotic* Bifidobacterium breve* CNCM I-4035 and its CFS have immunomodulatory effects in human intestinal-like dendritic cells. In particular, CFS decreased proinflammatory cytokines and chemokines in human intestinal dendritic cells challenged with* Salmonella enterica* serovar* Typhi* [51]. These results are particularly intriguing as they point out that whole bacterial cells or their metabolites can have opposite effects.

Considering the anatomy of the intestinal tract, it is known that microorganisms reside in the outer mucous layer and rarely they are able to reach the epithelium and underlying immune cells. Instead, the metabolites produced by probiotic in the lumen pass the intestinal barrier and interact with intestinal epithelial cells and macrophages. This process clarifies our choice of using the supernatants of different probiotics rather than the living or inactivated bacteria commonly investigated [40].

The intestinal inflammation that leads to the damage of the epithelium is partially associated with the production of oxygen radicals by neutrophils. This phenomenon clarifies the renewed interest in the search for new sources of antioxidants, which can be safely used in food. Among these, probiotics have been considered as an emerging source of effective antioxidants.

The antioxidative property of probiotics has been the subject of many studies in recent times [52, 53]. Some lactobacilli, used in the diet or as supplements, are known for their antioxidant effects [54]. Moreover, it has been reported that some probiotics result in increased activity of antioxidative enzymes or modulation of circulatory oxidative stress, protecting cells against carcinogen-induced damage [52]. Some authors hypothesize that probiotic bacteria exert their defensive effects against alcohol-induced oxidative stress in an animal model of alcoholic liver disease [55]. In this work, we tested whether probiotics CFS could reduce oxidative damage and free radical scavenging rate. Two assays were performed to evaluate the antioxidative activity of CFS of probiotics: an* in vitro* cell-free assay and a cell test that measured the luminol-enhanced chemiluminescence produced by* ex vivo* human neutrophils stimulated with phorbol 12-myristate 13-acetate. Neutrophils are short-lived myeloid cells that produce reactive oxygen species superoxide via the respiratory burst mechanism as a part of the defence response to infection [56]. In our study, CFS showed scavenging activity and they were able to contrast the oxidative response of neutrophils under inflammation.

## 5. Conclusions

Duary et al. [57] affirmed that probiotics should be explored either prophylactically or as biotherapeutics to manage inflammatory gut disorders, providing a safe and cost-effective complementary or alternative option to drug treatment.

Our results, indeed, show that probiotic metabolites, exhibiting anti-inflammatory and antioxidant effects, can be considered a suitable alternative approach for the formulation of probiotic complements. In particular,* L. acidophilus* and* L. casei* are able to downregulate the TNF-*α* secretion and upregulate the anti-inflammatory IL-10 production. Moreover,* L. casei* metabolites prevent IL-1*β* activation induced by LPS.

In conclusion, this work has demonstrated that probiotic metabolites exhibit a good anti-inflammatory and antioxidant property acting first on intestinal epithelial cells and then on immune cells; however, not all probiotics exert the same immunomodulatory action on the host, suggesting that the choice of probiotic strains used in nutraceutical formulations requires special attention.

Considering the complete lack of adverse effects, we believe that the incorporation of probiotics in foods could provide a good strategy for the production of functional foods as antioxidant and anti-inflammatory diet supplements, opening new prospects for their possible use for the treatment of human intestinal inflammation.

## Data Availability

All datasets analyzed during the current study are available from the corresponding author upon reasonable request.
